# Effects of Age and Body Mass Index on Thoracolumbar Spine X-Ray for Diagnosing Osteoporosis in Elderly Women: Tianliao Old People (TOP) Study 07

**DOI:** 10.1371/journal.pone.0161773

**Published:** 2016-09-08

**Authors:** Yin-Fan Chang, Chin-Sung Chang, Mei-Wen Wang, Chun-Feng Wu, Chuan-Yu Chen, Hsuan-Jui Chang, Po-Hsiu Kuo, Chih-Hsing Wu

**Affiliations:** 1 Department of Family Medicine, National Cheng Kung University Hospital, Tainan, Taiwan; 2 Department of Radiology, Tainan Hospital Xinhua Branch, Department of Health, Tainan, Taiwan; 3 Department of Family Medicine, Kuo General Hospital, Tainan, Taiwan; 4 Dynasty clinic, Tainan, Taiwan; 5 Department of Family Medicine, Changhua Christian Hospital, Changhua, Taiwan; 6 Department of Public Health & Institute of Epidemiology, College of Public Health, National Taiwan University, Taipei, Taiwan; Harvard Medical School/BIDMC, UNITED STATES

## Abstract

**Purpose:**

The aim of this study was to determine the effects of diagnostic discordance with or without a thoracolumbar spine lateral view X-ray in patients with osteoporosis.

**Methods:**

We randomly enrolled 368 women over 65 years old (74.3 ± 6.0 years) from Tianliao Township in 2009 (response rate: 75.7%). A diagnosis of osteoporosis was confirmed using one of these criteria: (1) a history of non-traumatic fracture, (2) vertebral fractures based on a thoracolumbar spine lateral view X-ray, or (3) a bone mineral density T-score ≤ -2.5 for the total hip, the femoral neck, the lumbar spine, or all 3 sites. The prevalence of osteoporosis in three groups was compared based on Model I (criteria 1+2) vs. Model II (criteria 1+3) vs. Model III (criteria 1+2+3). The role of thoracolumbar X-ray reflected by the diagnostic discordance of osteoporosis between Models II and III was evaluated.

**Results:**

The overall prevalence of osteoporosis was 78.3% (Model III, age-standardized 78.1%). The diagnostic discordance was 17.4% in the 368 participants. A logistic regression model showed that age was negatively associated with diagnostic discordance (odds ratio [OR] = 0.93, 95% confidence interval [CI]: 0.88–0.98, p < 0.05), but body mass index was positively associated (OR = 1.07, 95% CI: 1.00–1.15, p < 0.05).

**Conclusions:**

A thoracolumbar spine lateral view X-ray should be added for women ≥ 65 years old or with a body mass index ≥ 25 kg/m^2^ to minimize the diagnostic discordance in osteoporosis, especially in highly endemic regions.

## Introduction

Over the past decades, the elderly (≥ 65 years old) population has dramatically increased. Consequently, age-associated diseases like osteoporosis have become a major public health problem [1,2]. Osteoporosis is estimated to affect more than 200 million women worldwide, and a 240% increase in the incidence of hip fracture in women is projected by 2050 [1]. Furthermore, 50% of these hip fractures are expected to occur in Asia [3]. For example, the incidence of hip fractures in Taiwan rose significantly (30%) between 1996 and 2002 [4]. Osteoporosis is rapidly becoming an important worldwide healthcare problem, especially in Asia.

Traditionally, a diagnosis of osteoporosis is based on bone mineral density (BMD) measured using dual energy X-ray absorptiometry (DXA). However, osteoporosis is a metabolic bone disease characterized by low bone mass and the microarchitectural deterioration of bone tissue [5]. The diagnosis of osteoporosis should be based not only on BMD, but also on the facts of osteoporotic fractures. In 2015, the National Osteoporosis Foundation (NOF) Clinician’s Guide [6] said that the diagnosis of osteoporosis is established by measuring BMD or by hip or vertebral fractures in adults who do not undergo major trauma. In fact, half of patients with hip fracture have had prior fragility fractures, including unrecognized vertebral fractures [7, 8]. Therefore, despite the fracture history, the International Osteoporosis Foundation (IOF) suggested using vertebral fracture assessment (VFA) equipment to improve the identification of unrecognized vertebral fractures [9, 10].

A history of fracture was most commonly used in clinical practice to discover a patient’s prior fractures. However, the discordance between clinically and radiologically diagnosed vertebral fractures has been reported in several studies [11–13]. Approximately two-thirds of vertebral fractures are asymptomatic and have been underestimated in clinical practice [12]. Therefore, VFA was required to minimize the diagnostic discordance in osteoporosis [9, 10].

Several consensus guidelines have argued the indications for VFA in women [6, 14–17]. The 2007 official position of the International Society for Clinical Densitometry (ISCD) suggests that women ≥ 70 years old with a low BMD or with a history of non-vertebral fractures were indicated for VFA [14]. The NOF Clinician’s Guide [6] suggests that VFA be used with patients who are ≥ 70 years old, 65 to 69 years old with a BMD T-score ≤ -1.5, or 50 to 64 years old with specific risk factors (e.g., fractures caused by low-level trauma, a historical height loss ≥ 1.5 inches, a prospective height loss ≥ 0.8 inches, or recent long-term glucocorticoid treatment). In contrast, guidelines in Canada, the UK, and Japan do not propose indications for VFA [15–17]. However, none of the literature discusses indications for VFA in Asia. Furthermore, few studies have discussed the threshold of the risk factors for diagnostic discordance, such as age and body mass index (BMI). We hypothesized that a thoracolumbar spine lateral view X-ray would minimize the diagnostic discordance in DXA-diagnosed osteoporosis. The aim of this study was to show the effects of diagnostic discordance with and without a thoracolumbar spine lateral view X-ray in osteoporosis. The threshold of the risk factors for diagnostic discordance, such as age and BMI, are also discussed.

## Methods

### Participants

Tianliao Township in southern Taiwan is served by only one healthcare unit: the Public Health Center. Household registration data from 2009 showed the total population as 8,359 (4,632 males, 3,727 females) and the elderly population (≥ 65 years) was 1,981 (23.7%) [18].

The sampling method is previously described [19]. In brief, 1,040 elderly women were randomly sampled in July 2009. After excluding the deceased (n = 4) and those no longer residing in Tianliao (n = 165), a total of 368 participants were enrolled in the current study. The response rate was 75.7% (368/486) [19]. Each participant provided written informed consent that was approved by the Institutional Review Board of National Cheng Kung University Hospital.

### Questionnaires

Each participant filled out a structured questionnaire with the help of administrative staff well-trained in conducting face-to-face interviews [19]. The questionnaires included four domains: (1) sociodemographic characteristics (age and education); (2) habitual behaviors (cigarette smoking, and alcohol and tea drinking) [20, 21]; (3) medical history (diabetes mellitus, hypertension, thyroid disease, rheumatoid arthritis, and osteoarthritis); (4) osteoporosis-related risk factors: their history of fracture, their parents’ history of hip fracture, and their history of steroid use. The clinical risk factors for osteoporosis are based on a fracture risk assessment tool (FRAX) [22]. Secondary osteoporosis was defined if the patient had a disorder strongly associated with osteoporosis, such as type I diabetes, untreated long-standing hyperthyroidism, or premature menopause (< 45 years old).

### Anthropometric data

Body height (cm) and weight (kg) were measured in light clothing without shoes using a calibrated spring balance with a built-in ruler. BMI was calculated as weight in kilograms divided by height in meters squared (kg/m^2^). According to the Asia-Pacific BMI cutoff, participants were categorized into three subgroups: normal weight: BMI ≤ 23; overweight: BMI = 23 to < 25; and obese: BMI ≥ 25 [23].

### Bone mineral density measurement

BMD was measured at the total hip, femoral neck, and lumbar spine (L1–L4) regions using DXA (QDR Explorer; Hologic, Sunnyvale, CA, USA) installed in a mobile bus (DC-325R; Daeyoung Medical Systems, Seoul, Korea). The precision for the BMD measurements were 1.42% (total hip), 1.51% (femoral neck), and 1.15% (lumbar spine). The least significant changes (LSCs) (lumbar spine) for each region were 3.93% (total hip), 4.19% (femoral neck), and 3.17% (lumbar spine) [19]. The precision for each score was within the requirements of the ISCD official position statement [24]. T-score for BMD was generated based on a data set from the Asian population.

### Vertebral fracture assessment

The thoracolumbar spine lateral view X-rays (T4 to L6) obtained from the mobile bus were interpreted blindly by a single radiologist. Vertebral fractures were classified using a combination of the Genant et al. [25] semiquantitative (SQ) method and morphometry: grade 1 = a reduction in vertebral height of 20–25%, grade 2 = a reduction of 26–40%, and grade 3 = a reduction of over 40%.

### Diagnosis of osteoporosis

The diagnosis of osteoporosis was confirmed using different criteria: (1) a history of non-traumatic fracture [5], (2) vertebral fractures based on a thoracolumbar spine lateral view X-ray [6], or (3) a BMD T-score ≤ -2.5 on total hip, femoral neck, lumbar spine and 3 sites [5,19,26]. The prevalences of osteoporosis in the three groups were compared based on Model I (criteria 1+2) [6,9–10] vs. Model II (criteria 1+3)[5] vs. Model III (criteria 1+2+3) [5,6,9–10]. Because of the gap between a patient’s history of vertebral fractures and the radiological images, diagnostic discordance was defined by the difference between Models II and III.

### Statistical analysis

SPSS 17.0 for Windows was used for all analyses. Demographic data is expressed as proportionate percentiles or mean ± standard deviation. Multiple logistic regression model analysis was used to determine the independence of age, BMI, their parents’ history of hip fracture, their history of steroid use, and secondary osteoporosis on osteoporosis and diagnostic discordance. Significance was set at *p* < 0.05 (two-tailed).

## Results

Of 368 participants, the mean age was 74.3 ± 6.0 years and the mean BMI was 24.9 ± 3.9 kg/m^2^ (Table 1). The discordance group was younger (mean age: 72.4 ± 5.3 vs. 74.7 ± 6.0) and more overweight (BMI: 26.1 ± 4.0 vs. 24.7 ± 3.9 kg/m^2^) than the non-discordance group, but less illiterate (81.3% vs. 90.1%) and it had a history of fewer previous fractures (0.0% vs. 13.8%).

**Table 1:** Demographic data of 368 elderly (≥ 65 years) women living in a rural community.

	Total	Non-discordance	Discordance^a^
	n = 368	n = 304	n = 64
	Mean ± SD^#^	Mean ± SD	Mean ± SD
Variables	n (%)	n (%)	n (%)
Age, years**	74.3 ± 6.0	74.7 ± 6.0	72.4 ± 5.3
Body weight, kg**	55.4 ± 9.8	54.4 ± 9.6	60.1 ± 9.8
Body height, cm**	148.8 ± 6.0	148.3 ± 6.1	151.3 ± 4.9
Body mass index, kg/m2*	24.9 ± 3.9	24.7 ± 3.9	26.1 ± 4.0
Married	239 (64.9)	192 (63.2)	47 (73.4)
Illiterate*	326 (88.6)	274 (90.1)	52 (81.3)
Clinical risk factors in osteoporosis^b^			
Previous fracture**	42 (11.4)	42 (13.8)	0 (0.0)
Hip fracture history of parents	19 (5.2)	15 (4.9)	4 (6.3)
Current smokers	1 (0.3)	1 (0.3)	0 (0.0)
Use of steroids	17 (4.6)	14 (4.6)	3 (4.7)
Rheumatoid arthritis	0 (0.0)	0 (0.0)	0 (0.0)
Secondary osteoporosis	36 (9.8)	29 (9.5)	7 (10.9)
Alcohol drinkers	1 (0.3)	1 (0.3)	0 (0.0)
Self- reported systemic disease			
Hypertension	198 (53.8)	157 (51.6)	41 (64.1)
Diabetes mellitus, type 2	59 (16.0)	45 (14.8)	14 (21.9)
Hyperlipidemia	36 (9.8)	33 (10.9)	3 (4.7)
Hyperuricemia	11 (3.0)	8 (2.6)	3 (4.7)
Cerebrovascular events	11 (3.0)	10 (3.3)	1 (1.6)
Thyroid diseases	8 (2.2)	7 (2.3)	1 (1.6)
Osteoarthritis	6 (1.6)	5 (1.6)	1 (1.6)
Renal diseases	6 (1.6)	5 (1.6)	1 (1.6)
Liver diseases	4 (1.1)	2 (0.7)	2 (3.1)

^#:^ mean ± SD in continuous variables, Student’s *t* test; percentage in categorical variables, χ^2^ test;

* *p* < 0.05;

** *p* < 0.005.

^a:^ Osteoporosis was diagnosed using three different consecutive models:

Model I: defined by fracture history or an X-ray of a vertebral fracture of the thoracolumbar spine.

Model II: defined by fracture history or bone mineral density on any part of the lumbar spine, femoral neck, or total hip with a T-score ≤ -2.5.

Model III: defined by fracture history, an X-ray of a vertebral fracture of the thoracolumbar spine, or bone mineral density on any part of the lumbar spine, femoral neck, or total hip with a T-score ≤ -2.5.

Diagnostic discordance was defined by the difference between Model II and Model III.

^b:^ Definition of clinical risk factors is based on fracture risk assessment tool (FRAX).

**Fig 1:**
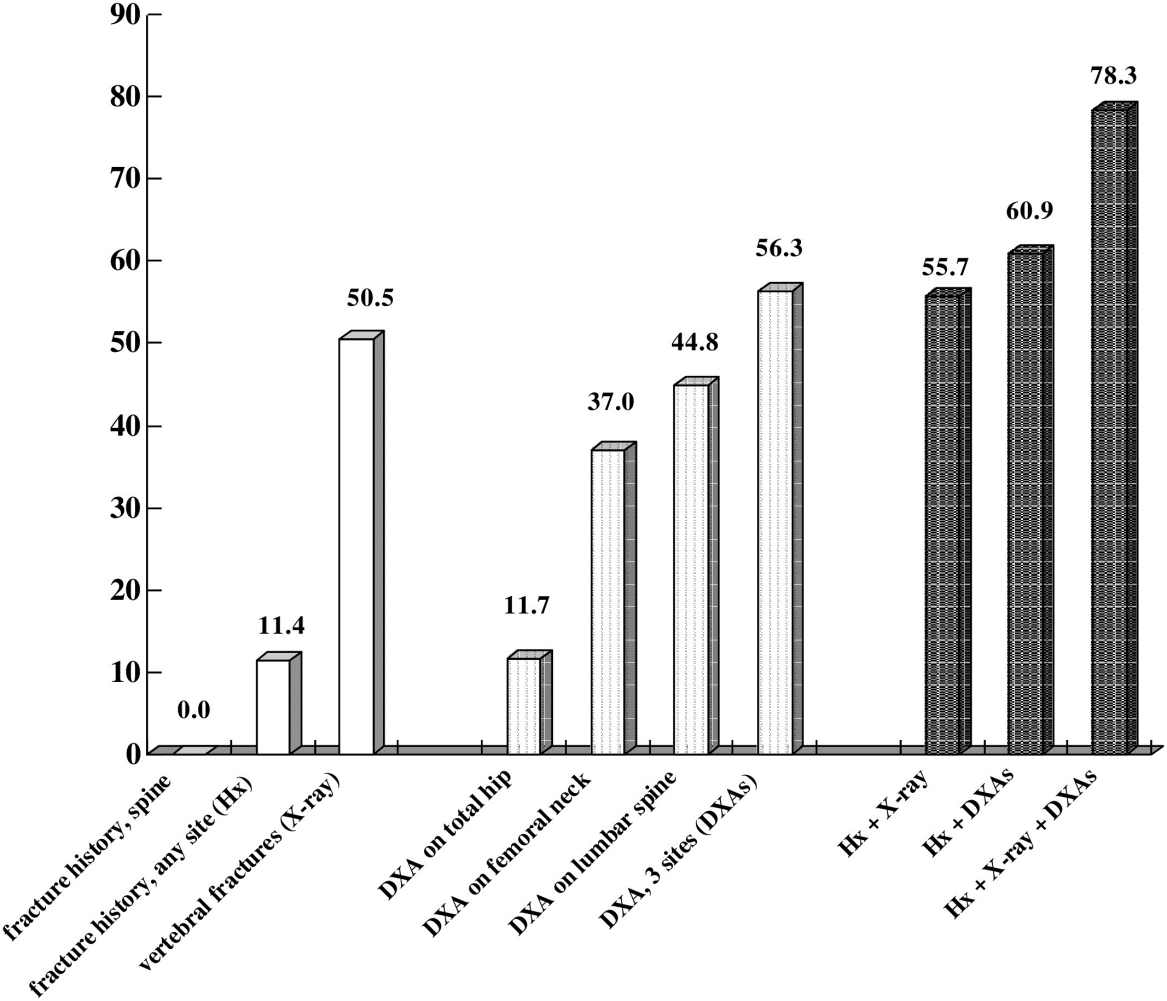
Prevalence of osteoporosis in 368 elderly women, measured using different methods. among 368 elderly women. The diagnosis of osteoporosis was confirmed using different criteria: (1) non-traumatic fracture history (**Hx**), (2) vertebral fractures based on a lateral view of a thoracolumbar spine X-ray (**X-ray**), or (3) a bone mineral density T-score ≤. -2.5 on either total hip, femoral neck, lumbar spine, or all 3 sites (**DXAs**). **Hx+X-ray:** the diagnosis of osteoporosis was based on criteria 1 or 2; **Hx+DXAs:** the diagnosis of osteoporosis was based on criteria 1 or 3; **Hx+X-ray+DXAs:** the diagnosis of osteoporosis was based on criteria 1, 2, or 3.

Of the 368 women > 65 years old, only 42 (11.4%) had a history of non-traumatic fracture, but none was a vertebral fracture. Vertebral fractures were identified using a lateral view of the thoracolumbar spine X-ray in 186 participants (50.5%) with a total of 394 fractures: 190 (48.2%) were grade 1, and 204 were grade 2 or 3. Fractures were most common at the thoracolumbar junction (T11 to L1). The mean BMDs were 0.704 ± 0.122 g/cm^2^ for total hip, 0.570 ± 0.101 g/cm^2^ for the femoral neck, and 0.743 ± 0.137 g/cm^2^ for the lumbar spine. Based on the BMDs in different regions, the prevalences of T-scores ≤ -2.5 were 11.7% (total hip), 37.0% (femoral neck), 44.8% (lumbar spine), and 56.3% (3 sites). The overall prevalences of osteoporosis were 55.7% (Model I), 60.9% (Model II), and 78.3% (Model III; age-standardized rate was 78.1%) (Fig 1).

Using a logistic regression model adjusted for age, BMI, their parents’ history of hip fracture, their history of steroid use, and secondary osteoporosis, the associations between osteoporosis and possible clinical risk factors are consecutively displayed in Models I-III (Table 2). Model I shows that only age (≥ 75 years) was positively associated with osteoporosis (OR = 1.75, 95% CI: 1.14–2.69, p < 0.05). Model II shows that age (≥ 75 years) was positively associated with osteoporosis (OR = 1.87, 95% CI: 1.19–2.94, p < 0.05), but that BMI was negatively associated (23.0–24.9 kg/m^2^, OR = 0.42, 95% CI: 0.22–0.78, p < 0.05; ≥ 25 kg/m^2^, OR = 0.33, 95% CI: 0.19–0.56, p < 0.001). Finally, Model III shows that age (≥ 75 years) was significantly positively associated with osteoporosis (OR = 2.06, 95% CI: 1.19–3.57, p < 0.05), but that BMI was negatively associated (23.0–24.9 kg/m^2^, OR = 0.33, 95% CI: 0.16–0.71, p < 0.05; ≥ 25 kg/m^2^, OR = 0.41, 95% CI: 0.21–0.81, p < 0.05).

**Table 2:** Multiple logistic regression models of osteoporosis risk factors in 368 elderly women living in a rural community.

		Diagnosis of osteoporosis		
		Model 1	Model II	Model III
		Odds ratio (95% CI)	Odds ratio (95% CI)	Odds ratio (95% CI)
Age, years	(< 75: 0, ≥ 75: 1)	1.75 (1.14–2.69)*	1.87 (1.19–2.94)*	2.06 (1.19–3.57)*
Body mass index, kg/m^2^				
< 22.9		1.000	1.000	1.000
23.0–24.9		0.64 (0.36–1.14)	0.42 (0.22–0.78)*	0.33 (0.16–0.71)*
≥ 25		1.03 (0.63–1.68)	0.33 (0.19–0.56)**	0.41 (0.21–0.81)*
Hip fracture history of parents	(No: 0, Yes: 1)	0.96 (0.38–2.45)	0.57 (0.22–1.49)	0.60 (0.22–1.69)
Use of steroids	(No: 0, Yes: 1)	1.79 (0.61–5.25)	1.32 (0.44–3.98)	1.78 (0.39–8.17)
Secondary osteoporosis	(No: 0, Yes: 1)	0.94 (0.47–1.89)	0.93 (0.45–1.94)	1.06 (0.45–2.48)

Dependent variable: osteoporosis (Model I-III);

* *p* < 0.05;

** *p* < 0.005.

Osteoporosis was diagnosed using three different consecutive models:

Model I: defined by fracture history or an X-ray of a vertebral fracture of the thoracolumbar spine.

Model II: defined by fracture history or bone mineral density on any part of the lumbar spine, femoral neck, or total hip with a T-score ≤ -2.5.

Model III: defined by fracture history, an X-ray of a vertebral fracture of the thoracolumbar spine, or bone mineral density on any part of the lumbar spine, femoral neck, or total hip with a T-score ≤ -2.5.

Diagnostic discordance was defined by the difference between Model II and Model III.

The percentage of diagnostic discordance was 17.4% (64/368). Using logistic regression adjusted for age, BMI, their parents’ history of hip fracture, their history of steroid use, and secondary osteoporosis, age was negatively associated with diagnostic discordance (OR = 0.93, 95% CI: 0.88–0.98, p < 0.05), while BMI was positively associated with diagnostic discordance (OR = 1.07, 95% CI: 1.00–1.15, p < 0.05) (Table 3). Using dichotomized variables for further exploring the threshold of age and BMI to discriminate the changes of diagnostic discordance, it identically showed that the diagnostic discordance increased dramatically in patients < 70 years and with a BMI ≥ 25 kg/m^2^ (Table 4).

**Table 3:** Multiple logistic regression models of the risk factors for diagnostic discordance^a^ of a diagnosis of osteoporosis, with or without a lateral view of a thoracolumbar spine X-ray, in elderly women living in a rural community (n = 64).

		Diagnosis Discordance
Risk Factors		OR (95% CI)
Age, years		0.93 (0.88–0.98)*
Body mass index, kg/m2		1.07 (1.00–1.15)*
Hip fracture history of parents	(No: 0, Yes: 1)	1.13 (0.36–3.61)
Use of Steroids use	(No: 0, Yes: 1)	1.04 (0.28–3.88)
Secondary osteoporosis	(No: 0, Yes: 1)	1.08 (0.44–2.64)

OR (95% CI): odds ratio (95% confidence interval),

* *p* < 0.05.

^a:^ Osteoporosis was diagnosed using three different consecutive models:

Model I: defined by fracture history or an X-ray of a vertebral fracture of the thoracolumbar spine.

Model II: defined by fracture history or bone mineral density on any part of the lumbar spine, femoral neck, or total hip with a T-score ≤ -2.5.

Model III: defined by fracture history, an X-ray of a vertebral fracture of the thoracolumbar spine, or bone mineral density on any part of the lumbar spine, femoral neck, or total hip with a T-score ≤ -2.5.

Diagnostic discordance was defined by the difference between Model II and Model III.

**Table 4:** Change in diagnostic discordance^a^ of osteoporosis, with or without a lateral view of a thoracolumbar spine X-ray, by age and body mass index in elderly women living in a rural community (n = 368).

	Osteoporosis Prevalence (%)		Diagnostic
Variables	DXA+Hx	DXA+Hx+X-ray	Discordance
Age, years			
< 70	44.6	70.3	25.7
< 75	53.1	72.5	19.4
< 80	56.1	76.1	20.0
< 85	59.3	77.2	17.9
Body mass index, kg/m^2^			
≥ 23	53.2	73.4	20.2
≥ 24	52.7	73.6	20.9
≥ 25	50.9	74.3	23.4
≥ 26	50.8	75.8	25.0
≥ 27	48.9	75.0	26.1
≥ 28	49.2	76.9	27.7

^a:^ Osteoporosis was diagnosed using three different consecutive models:

Model I: defined by fracture history or an X-ray of a vertebral fracture of the thoracolumbar spine.

Model II: defined by fracture history or bone mineral density on any part of the lumbar spine, femoral neck, or total hip with a T-score ≤ -2.5.

Model III: defined by fracture history, an X-ray of a vertebral fracture of the thoracolumbar spine, or bone mineral density on any part of the lumbar spine, femoral neck, or total hip with a T-score ≤ -2.5.

Diagnostic discordance was defined by the difference between Model II and Model III.

## Discussion

We found that the prevalence of osteoporosis in elderly women based on 3 DXA scan sites (lumbar spine, total hip, and femoral neck) was 57.7%. In the United States, around 30% of postmenopausal white women based on femoral neck DXAs were estimated to have osteoporosis [27], as were approximately 21% of European women 50–84 years old [28]. Consistently, the prevalences of osteoporosis in women 50–79 years old based on the 3 DXA sites were 38.0% at the lumbar spine, 11.6% at the femoral neck, and 15.3% at the total hip in Japan [29] and in women > 50 years old was 35.5% in Korea [30]. Comparably, the Nutrition and Health Survey in Taiwan (NAHSIT 2005–2008) reported that the prevalences were 50.3% in women ≥ 60 years old, and 63.7% in women ≥ 70 years old [2]. Taiwan’s osteoporosis prevalences are higher than those in other countries, which might indirectly explain why Taiwan has the highest hip fracture incidence in Asia [31]. Moreover, it is more prudent to use all 3 sites than only a single DXA site to identify patients with osteoporosis.

We also found that diagnostic discordance with or without a thoracolumbar X-ray in elderly women was 17.4%. Osteoporosis is a metabolic bone disease characterized by low BMD and microarchitectural deterioration of bone tissue [5]. BMD explains only 50–60% of fracture risk. Other factors, such as bone quality and falling affect the fracture probability. Therefore, diagnostic discordance is reasonable in terms of a DXA-diagnosed osteoporosis. A Canadian study [32] reported that adding VFA to BMD altered the diagnostic classification in approximately 1 out of every 5 patients, and a Dutch study [33] said that adding VFA to BMD increased treatment requirements by 23%. It is crucial to evaluate the treatment requirements of a patient with osteoporosis, whether or not the patient has a fracture. For example, according to the Taiwan version of FRAX, the ten-year hip fracture probability of a 65-year-old overweight woman (BMI = 25 kg/m^2^) with or without fractures was 4.4% (high fracture risk) vs. 1.8% (moderate fracture risk). Therefore, with or without a VFA makes a difference not only in the diagnosis but also in the treatment and prognosis of osteoporosis. On the other hand, vertebral fractures were most common at the thoracolumbar junction (T11 to L1) [10] and the DXA scans performed in our study did not analyze the T11 and T12 vertebrae. The diagnostic discordance could potentially be lower if participants with fractures at T11 and T12 had an initial DXA scan to show a low BMD at the corresponding segments. However, the gold standard of diagnosis in osteoporosis is DXA scans at the total hip, femoral neck, and lumbar spine (L1–L4) regions, not including the thoracic spine. Therefore, the lateral view of thoracolumbar spine X-ray should be added to minimize the diagnostic discordance in osteoporosis.

Most hospitals and clinics fail to capture the first fracture, especially the vertebral fracture. Over 80% of fracture patients are never screened or treated for osteoporosis [34]. No wonder the IOF announced the “STOP AT ONE” campaign for secondary prevention of osteoporotic fractures and emphasized doing a clinical assessment, including BMD and fracture risk assessment, especially for people with a history of non-traumatic fracture. Therefore, the UK Department of Health developed a fracture liaison service to identify patients with fractures and to provide post-fracture care. However, there was no consistent strategy to discover unrecognized vertebral fractures in the UK-based fracture liaison service [35]. In our study, because a lateral view of the thoracolumbar spine X-ray identified 50% of patients without a history of non-traumatic fracture as having vertebral fractures, using a VFA might be a feasible and adequate strategy for the fracture liaison service to discover unrecognized vertebral fractures.

The ISCD official position statement suggests that women > 70 years old with low BMD or between 60 and 69 years old with non-vertebral fractures should undergo VFA to radiologically screen for vertebral fractures to increase the certainty of a diagnosis of osteoporosis [7]. A Moroccan study [36] enlarged the indications of VFA in the presence of risk factors such as age over 60, multiparity, a history of peripheral traumatic fractures, and a low BMI. Our study further explored the threshold of the risk factors for diagnostic discordance with and without a thoracolumbar spine X-ray. Therefore, in a high endemic region, a cost-effective strategy for diagnosing osteoporosis might be adding VFA, i.e., the lateral view of a thoracolumbar spine X-ray, to DXA, especially in women between 65 and 70 years old with a BMI ≥ 25 kg/m^2^. Overweight and obesity related to a lower 25-hydroxy vitamin D test score and to vitamin D’s being hidden in fat-soluble adipose tissue [37, 38]. Furthermore, increased BMI reduced muscle composition and resulted in so called “sarcopenic obesity”, a combination of obesity and low muscle mass, which might increase the risk of falls and fractures [39, 40].

Our study has several limitations. Firstly, our participants were recruited from a rural community. Their life styles, nutritional status, and physical activity might differ from those of people who live in more urban communities. However, the prevalence of osteoporosis is compatible with the survey in the 2005–2008 Nutrition and Health Survey in Taiwan [2], a representative sample of people residing in different geographical regions in Taiwan; thus, our findings might be able to be extrapolated to other high endemic regions. Secondly, our study was focused on ambulatory patients. Because most of those who did not respond to our request to enroll them in our study were older than those who did, our findings might underestimate the prevalence of osteoporosis. Because of the low awareness of osteoporosis, only 3 participants had been prescribed anti-osteoporosis medication and only 54 (14.7%) had been prescribed a calcium supplement [19]. Underdetection, underdiagnosis, and undertreatment might further increase the risk of fragility fractures, which cannot be overemphasized, especially in highly endemic regions [34].

In conclusion, the lateral view of the thoracolumbar spine X-ray should be added in women ≥65 years old or with a BMI ≥ 25 kg/m^2^ to minimize the diagnostic discordance in DXA-diagnosed osteoporosis, especially in highly endemic regions.
